# Prediction of tumor regression grading in rectal cancer neoadjuvant chemoradiotherapy: a habitat radiomics analysis of imaging biomarker

**DOI:** 10.1186/s12880-026-02397-x

**Published:** 2026-05-05

**Authors:** Xue Sha, Xue Dou, Luping Ma, Qingtao Qiu, Zhenkai Li, Tengxiang Li, Yongbin Cui, Huazhong Shu, Yong Yin

**Affiliations:** 1 Department of Radiation Oncology, Shandong Cancer Hospital and Institute, Shandong First Medical University and Shandong Academy of Medical Sciences, No. 440 Jiyan Road, Huaiyin District, Jinan, 250117 China; 2 Department of Radiation Medicine, Shandong First Medical University and Shandong Academy of Medical Sciences, Taian, China; 3 School of Computer Science and Engineering, Southeast University, No. 2 Sipailou Road, Xuanwu District, Nanjing, 210096 China

**Keywords:** Rectal cancer, Tumor regression grading, Neoadjuvant chemoradiotherapy, Pathologic complete response, Habitat radiomics

## Abstract

**Background:**

Tumor regression grading (TRG) is a core prognostic predictor of treatment outcomes in rectal cancer. Conventional TRG assessment methods are limited in capturing the full complexity of intratumoral heterogeneity. Advances in medical imaging, particularly radiomics and habitat-based analysis, hold promise the improve TRG prediction by quantitatively characterizing subregional tumor features. This study aimed to evaluate the performance of habitat radiomics in preoperatively predicting TRG in rectal cancer patients receiving neoadjuvant chemoradiotherapy (nCRT).

**Methods:**

Computed tomography (CT) images were analyzed to compare the predictive performance of conventional radiomics features and habitat-based analysis. Tumor regions of interest (ROIs) were segmented, extracting local imaging features. Voxel-level clustering was employed to identify distinct intratumoral subregions. Machine learning algorithms, including ExtraTrees, support vector machine (SVM), and Random Forest, were applied to predict TRG.

**Results:**

For the conventional radiomics model, the ExtraTrees algorithm yielded superior performance, with AUCs of 0.912 and 0.817 in training and testing cohorts, respectively, outperforming SVM and Random Forest. The habitat model outperformed conventional radiomics model, while the combined model integrating habitat features and clinical variables yielded the optimal efficacy (training AUC = 0.916, test AUC = 0.833). In the binary classification task of TRG0 (pathologic complete response, pCR) vs. TRG1–2, the Habitat model achieved a test AUC of 0.884, and the combined model further reached 0.929. SHAP analysis identified that features from the H1 subregion and wavelet-transformed features were the top predictive contributors.

**Conclusion:**

Habitat-based radiomics, especially when integrated with clinical data, significantly improves the preoperative prediction of TRG in rectal cancer patients undergoing nCRT, providing a powerful tool to advance personalized oncology. Further validation in large-scale, multicenter, independent cohorts is warranted to facilitate the clinical translation of this approach.

**Supplementary Information:**

The online version contains supplementary material available at 10.1186/s12880-026-02397-x.

## Introduction

The standard treatment paradigm for locally advanced rectal cancer (LARC) consists of neoadjuvant chemoradiotherapy (nCRT) followed by radical surgery, and total neoadjuvant therapy (TNT) is recommended for patients with high-risk features [1, 2]. nCRT has been proven to improve R0 resection rates, reduce local recurrence, and increase the possibility of sphincter preservation, highlighting the critical importance of comprehensive pretreatment evaluation and precise risk stratification for individualized treatment optimization [3, 4]. Approximately 15–30% of LARC patients achieve pathologic complete response (pCR) after nCRT, which is closely associated with favorable long-term survival outcomes [5]. For these patients, an organ-preserving “watch-and-wait” strategy may be a feasible alternative to radical surgery, effectively improving quality of life without compromising oncological safety.

Tumor regression grading (TRG) serves as a standardized system to classify histopathologic tumor response to neoadjuvant therapy, which provides critical information for prognosis, therapeutic efficacy assessment, and postoperative clinical decision-making [6]. Accurate preoperative TRG prediction not only enables non-invasive assessment of neoadjuvant treatment response, but also can be integrated with postoperative histopathological characteristics to construct a risk stratification system. This system can stratify LARC patients into low- and high-risk groups for disease-free survival (DFS) and overall survival (OS), thereby optimizing postoperative adjuvant treatment decision-making, improving tumor staging prediction performance, and ultimately advancing the goal of precision oncology for rectal cancer. Despite its well-recognized clinical value, TRG assessment still faces several unmet challenges in clinical practice, including inconsistent grading criteria across systems, low interobserver agreement, frequent discrepancies between clinical complete response (cCR) and pCR, and heterogeneous intratumor response patterns [7]. In the era of precision medicine, accurate preoperative prediction of TRG is essential to optimize individualized neoadjuvant treatment strategies for rectal cancer, balancing the dual goals of maximizing patient survival and preserving quality of life.

Conventional radiomics can extract high-dimensional quantitative imaging features,, and has shown potential in predicting treatment response and prognosis in multiple malignancies [8, 9]. However, traditional radiomics mainly focuses on global features of the whole tumor volume, which often fails to fully capture the complex spatial heterogeneity of tumor microenvironment (TME) [10–12]. To address this limitation, this study integrated habitat-based analysis with conventional radiomics to improve the predictive performance of TRG in rectal cancer. Habitat radiomics performs voxel-level clustering to identify distinct intratumoral subregions with unique biological characteristics, and extracts quantitative features from each subregion separately [13]. This method combines global and local heterogeneity features, overcoms the limitation of traditional radiomics that only capture average tumor characteristics, and can more accurately reflect the biological heterogeneity of rectal cancer.

Previous studies have confirmed that DWI-MRI-derived intratumoral heterogeneity features based on intratumoral ecological diversity can capture key tumor microenvironment characteristics, improve tumor downstaging prediction in LARC patients receiving neoadjuvant chemoradiotherapy, and guide individualized patient selection. However, CT-based habitat model established in this study has better clinical scalability, with wider clinical availability, no absolute contraindications, and lower examination costs. This innovative approach enabled us to construct a TRG prediction model that synergistically integrates radiomic and habitat-derived features, indicating that habitat-based analysis provides critical prognostic information beyond the capacity of traditional radiomics. This integrative strategy is expected to improve the predictive accuracy of TRG, and holds great promise for optimizing individualized treatment planning for LARC patients.

## Methods

### Study Population and Datasets

**Fig. 1:**
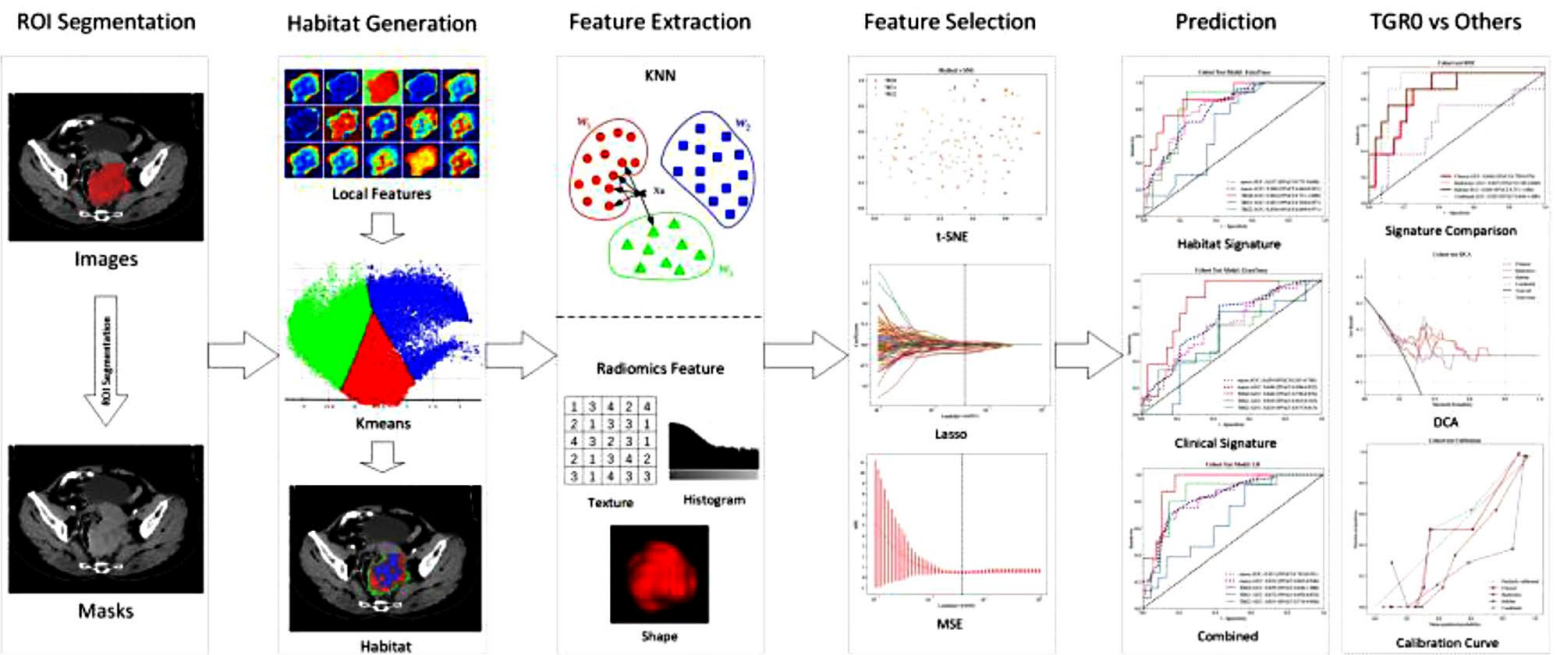
Workflow of the habitat-based radiomics model for predicting TRG in rectal cancer, including ROI segmentation, habitat generation, feature extraction, feature selection, model construction, and performance validation

This retrospective study was conducted in accordance with the Declaration of Helsinki and approved by the Institutional Review Board of participating hospital. The requirement for written informed consent was waived due to the retrospective design and anonymized use of patient data. The study dataset consisted of patients with histologically confirmed rectal adenocarcinoma who received standard nCRT followed by radical surgery at our institution between March 2018 and September 2024. The inclusion criteria were as follows: (a) histologically confirmed primary rectal adenocarcinoma; (b) completed standard nCRT and subsequent radical surgery; (c) available definitive postoperative TRG assessment in the pathology report; (d) age between 28 and 80 years at diagnosis; (e) no prior antitumor treatment before baseline CT examination; (f) underwent radical resection with curative intent. The exclusion criteria were: (a) history of other malignancy within the past 5 years; (b) incomplete postoperative imaging or clinical follow-up data for recurrence assessment. The overall workflow of the habitat radiomics model is illustrated in Fig. 1, including region of intrest (ROI) segmentation, habitat clustering, feature extraction, feature selection, model construction for TRG prediction, and performance validation.

### Image acquisition and preprocessing

All patients underwent contrast-enhanced abdominopelvic CT examination using a 64-slice multidetector CT scanner (LightSpeed16, Philips Healthcare, Amsterdam, Netherlands) within one week before the initiation of nCRT. The scanning parameters were as follows: tube voltage 120 kVp, tube current 200–250 mAs with automatic exposure control, slice thickness 3 mm, reconstruction interval 1 mm, pitch 1.0, and a standard soft tissue reconstruction algorithm. Non-ionic contrast agent (iopromide, 370 mg I/mL, Bayer Schering Pharma, Berlin, Germany) was intravenously injected at a rate of 3.0 mL/s with a total dose of 1.5 mL/kg body weight, and the portal venous phase scan was performed 60 s after contrast injection.

All CT images were preprocessed using Python (version 3.7.12) with the SimpleITK library (version 2.2.1) and OpenCV library (version 4.6.0) following the standard preprocessing pipeline: (1) resampling: images were resampled to an isotropic voxel spacing of 1 × 1 × 1 mm³ using B-spline interpolation via SimpleITK; (2) Intensity normalization: z-score normalization was performed on voxel intensity within the ROI using scikit-learn (version 1.0.2); (3) Windowing: the window width and level were set to 250 and 40 HU, respectively, using OpenCV, and the voxel intensity was clipped to the range of [-85, 165] HU to standardize the grayscale distribution across all images.

### Intratumor heterogeneity (ITH) signature construction

#### ROI segmentation

Three-dimensional (3D) ROIs covering the entire visible primary tumor lesion were manually delineated slice by slice by two experienced abdominal radiologists (8 and 10 years, respectively) using MIM Maestro software (v6.8.2, Cleveland, OH, USA), ensuring precise and reliable segmentation. For regions with inconsistent segmentation between the two readers, a consensus reading was performed by a senior radiation oncologist with 15 years of experience in rectal cancer management to obtain the final unified ROI. The Dice similarity coefficient (DSC) of 0.88 ± 0.05 confirmed high nterobserver consistency and reliability of the segmentation results. Detailed DSC results are presented in Supplementary Materials 2.

#### Habitat region generation

Intratumoral subregions (habitats) were generated using a voxel-wise habitat analysis framework, which was constructed based on quantitative local imaging descriptors derived from CT images. A 5 × 5 × 5 sliding window was applied across each volume of interest (VOI) to calculate 19 radiomic features for each voxel, including first-order statistical features (e.g., entropy, mean absolute deviation) and texture metrics. The 19 voxel-level features were selected based on three core principles: clinical relevance, imaging feasibility and dimensionality balance. The complete list of the 19 features used for constructing the high-dimensional local feature map is provided in Supplementary Materials 1.

This process generated a high-dimensional local feature map for each tumor VOI. Voxel-level feature vectors were then clustered into 2–10 subregions using the unsupervised K-means clustering algorithm. The optimal number of clusters was determined by the Calinski–Harabasz index, which simultaneously evaluates intra-cluster compactness and inter-cluster separability. Thw detailed process of tumor habitat generation was provided Supplementary Materials 1.

#### Feature extraction and selection

Radiomic features, including morphological, intensity, and texture descriptors, were extracted from habitat-derived subregions, as well as the whole tumor volume for the conventional radiomics model. Morphological features characterized the geometric properties of the lesions; first-order intensity features quantified the distribution of voxel signal values; and texture features captured intratumoral heterogeneity through multiple matrix-based analytical approaches. For subregions without representative cluster centers from unsupervised clustering, the missing feature rate in our study was < 0.1%, which was imputed using the KNN algorithm m. All feature extraction was performed using the PyRadiomics library (version 3.0.1), in strict compliance with the Imaging Biomarker Standardization Initiative (IBSI) standards.

All extracted features were Z-score normalized, then sequentially screened via univariate analysis (*p* < 0.05), Pearson correlation analysis (excluding features with |r| > 0.9 or low variance), minimum Redundancy Maximum Relevance (mRMR) ranking, and Least Absolute Shrinkage and Selection Operator (LASSO) regression with 10-fold cross-validation to obtain the final feature set for model construction.

### Predictive model construction

Predictive models for the Radiomics Signature, ITH (Habitat) Score and Clinical Signature were constructed using the LASSO-refined feature sets as inputs for machine learning classifiers. Hyperparameters of all models were optimized using grid search and 5-fold cross-validation in the training cohort. To address class imbalance, the Synthetic Minority Over-sampling Technique (SMOTE) was applied according to the proportion of samples with different TRG labels.

- **Conventional Radiomics Model**: Selected radiomic features from the whole tumor volume were used to construct modles using three machine learning algorithms: SVM (to leverage its robustness in high-dimensional, linearly separable data), Random Forest and ExtraTrees (ensemble-based methods to capture complex non-linear relationships and high-order feature interactions).
- **Habitat Model**: The habitat model was constructed using features extracted from distinct intratumoral subregions, integrated via a multi-habitat fusion strategy. Except for the differences in input feature type, the feature selection and model construction procedures were completely consistent with those of the conventional radiomics model to ensure methodological uniformity.
- **Clinical Model**: Available baseline clinical variables were included for model development. Significant clinical predictors were identified via univariate and multivariate analysis, and then modeled using the same machine learning algorithms as the radiomics and habitat models.
- **Combined Model**: To furtherimprove predictive performance, the predicted probability outputs of the clinical model and the habitat model were integrated via logistic gegression, resulting to construct a comprehensive combined model, which effectively synthesized clinical and imaging-derived intratumoral heterogeneity features.

### Model validation and interpretability

The discriminative performance of all models was evaluated across three classification tasks: (1) binary classification of TRG0 (pCR) vs. TRG1-2 (non- pCR); (2) binary classification of TRG0-1 (good response) vs. TRG2 (poor response); (3) multiclass classification of TRG0, TRG1 and TRG2. Among them, the TRG0 vs. non-pCR task was the key focus of this study, consistent with clinical concerns for pCR prediction in rectal cancer.

Model performanc was quantified using accuracy (Acc), AUC, sensitivity, specificity, positive predictive value (PPV), and negative predictive value (NPV). The DeLong test was used to compare the differences in AUC between different models. Model calibration was evaluated using the Hosmer–Lemeshow (HL) test, and the clinical net benefit was assessed via Decision Curve Analysis (DCA).

To enhance interpretability, t-distributed Stochastic Neighbor Embedding (t-SNE) was used for exploratory dimensionality reduction and visualization of the high-dimensional radiomic/habitat feature space, to intuitively show the sample distribution characteristics of different tumor regression grading (TRG) subgroups. SHapley Additive exPlanations(SHAP) values were calculated to quantify the contribution of each feature to the model’s predictive outcomes, and to rank the importance of predictive features.

### Statistical analysis

The normality of continuous clinical features was assessed using the Shapiro- Wilk test. Normally distributed continuous variables were presented as mean ± standard deviation, and comparisons between cohorts were performed using one-way Analysis of Variance (ANOVA). Categorical variables were presented as numbers (percentages), and comparisons between groups were performed using the chi-square (χ²) test. A two-sided *p* < 0.05 was considered statistically significant. All statistical analyses, as well as the development and evaluation of machine learning models, were performed using Python (version 3.7.12), Onekey (version 3.3.5), and scikit-learn (version 1.0.2).

## Results

### Baseline patient characteristics

A total of 121 eligible rectal cancer patients were finally included in this study, and their baseline clinical characteristics were extracted from the electronic medical record system. The entire cohort was randomly divided into a training cohort (70% of patients, *n* = 85) and an internal testing cohort (30% of patients, *n* = 36). To minimize selection bias, repeated random partitioning was conducted until no significant differences in baseline clinical variables were observed between the two cohorts (*p* > 0.05). The baseline characteristics of the two cohorts are summarized in Table 1.

**Table 1:** Baseline clinical characteristics of the training and testing cohorts

Feature_name	ALL	Training	Testing	*P* value
Age (years)	58.30 ± 10.69	57.95 ± 11.04	59.11 ± 9.94	0.588
Tumor length (cm)	4.93 ± 2.20	5.07 ± 2.32	4.58 ± 1.87	0.334
Sex, n (%)				0.052
0	81(66.94)	62(72.94)	19(52.78)	
1	40(33.06)	23(27.06)	17(47.22)	
Clinical T stage, n (%)				0.136
0	3(2.48)	2(2.35)	1(2.78)	
1	7(5.79)	3(3.53)	4(11.11)	
2	76(62.81)	51(60.00)	25(69.44)	
3	35(28.93)	29(34.12)	6(16.67)	
Lymph node status, n (%)				0.231
0	15(12.40)	8(9.41)	7(19.44)	
1	63(52.07)	44(51.76)	19(52.78)	
2	43(35.54)	33(38.82)	10(27.78)	
Location, n (%)				0.694
0	54(44.63)	36(42.35)	18(50.00)	
1	61(50.41)	45(52.94)	16(44.44)	
2	6(4.96)	4(4.71)	2(5.56)	
Preoperative serum CEA, n (%)				0.785
0	87(71.90)	60(70.59)	27(75.00)	
1	34(28.10)	25(29.41)	9(25.00)	

### Tumor habitat clustering, feature extraction, and selection

#### Optimal number of clusters

**Fig. 2:**
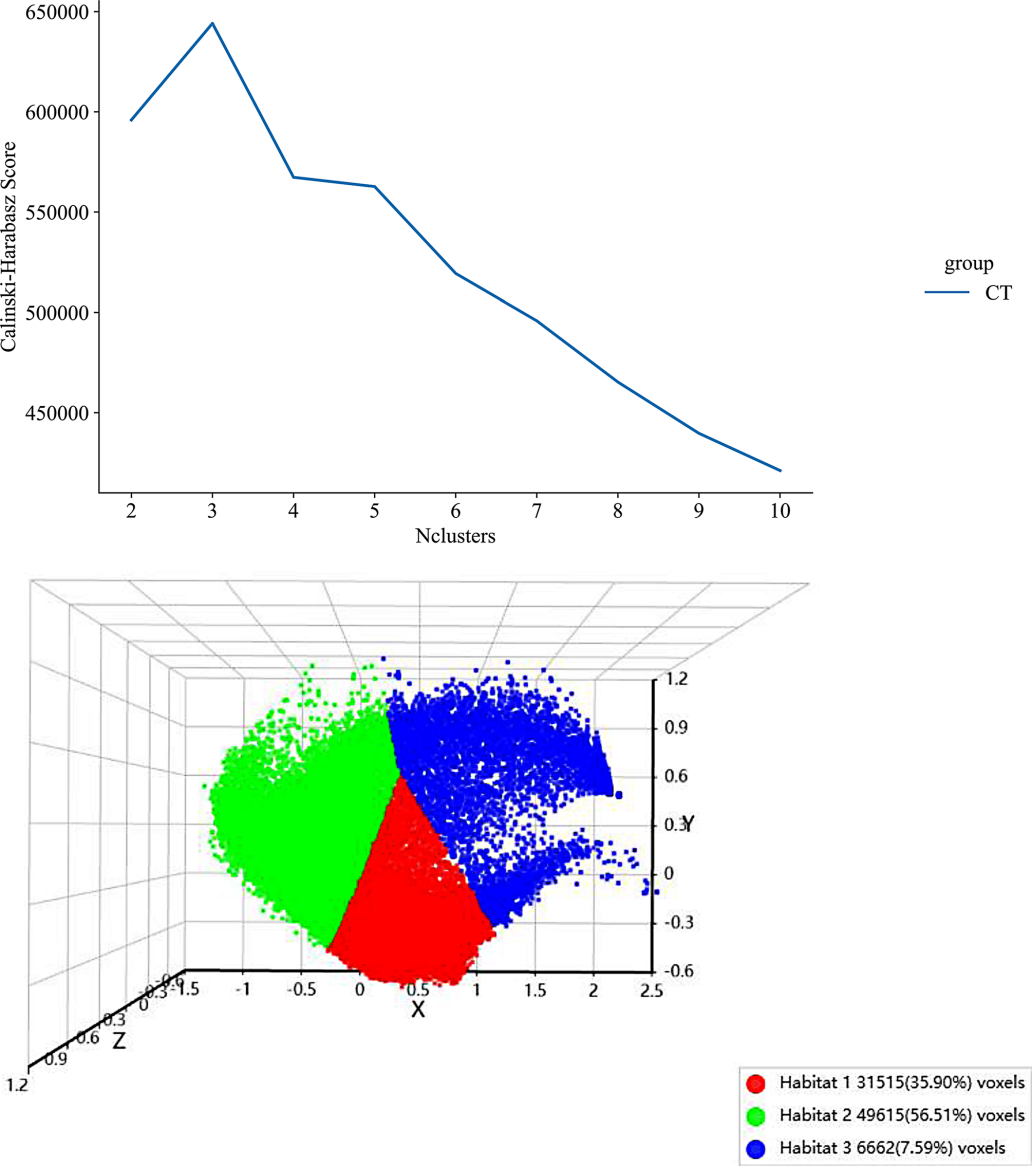
**a**, Calinski–Harabasz scores for different numbers of clusters; **b**, 3D feature distribution of three habitats in a representative patient (median tumor size, TRG2) from the training cohort, with color indicating normalized voxel values and volume ratios of each habitat shown in the lower right corner

Clustering performance was evaluated for 2–10 clusters using the Calinski–Harabasz index, which showed an initial increasing trend followed by a decreasing trend, with the peak value observed at 3 clusters. Accordingly, 3 intratumoral subregions were selected as the optimal solution for subsequent habitat analysis. The clustering process and results are illustrated in Fig. 2.

**Fig. 3:**
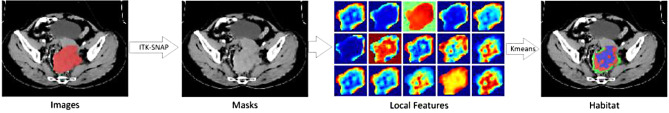
Representative axial CT image showing three distinct biological tumor habitats in a rectal cancer patient

Consistent with the biological heterogeneity of solid tumors, the three intratumoral subregions correspond to distinct habitats with unique radiological and biological characteristics:.Habitat 1 (H1, red) corresponds to the avascular necrotic component of the tumor with low CT attenuation; Habitat 2 (H2, green) represents peritumoral fibrotic stroma with moderate CT attenuation; Habitat 3 (H3, blue) denotes the highly proliferative tumor core with high CT attenuation. Representative examples of the clustered habitats within tumor volumes are shown in Fig. 3.

#### Radiomic feature extraction and selection

A total of 1,925 handcrafted radiomic features were extracted from each VOI, which were categorized into three domains: shape (14 features), first-order intensity statistics (360 features), and texture descriptors (the remaining features). After LASSO regression with 10-fold cross-validation to determine the optimal λ (λ = 0.023), a total of 27 non-zero coefficient features were retained for the habitat model, and 19 non-zero coefficient features were retained for the conventional radiomics model. For the retained habitat model features, 11 were texture features (40.7%), 9 were intensity features (33.3%), 5 were morphological features (18.5%), and 2 were wavelet-transformed composite features (7.4%); the retained conventional radiomics model features were mainly composed of texture features (12, 63.2%) and intensity features (7, 36.8%), with no morphological features retained.Habitat Model Performance and SHAP Interpretability Analysis.

Among habitat-based models, ExtraTrees achieved the highest discriminative performance, with AUCs of 0.912 (95% CI: 0.875–0.949) in the training cohort and 0.817 (95% CI: 0.737–0.896) in the test cohort, outperforming SVM (0.869/0.711) and Random Forest (0.853/0.582). These results demonstrate that ExtraTrees robustly captures intratumoral heterogeneity and provides superior generalization compared with other algorithms. Details of the habitat model are summarized in Table S1.

**Fig. 4:**
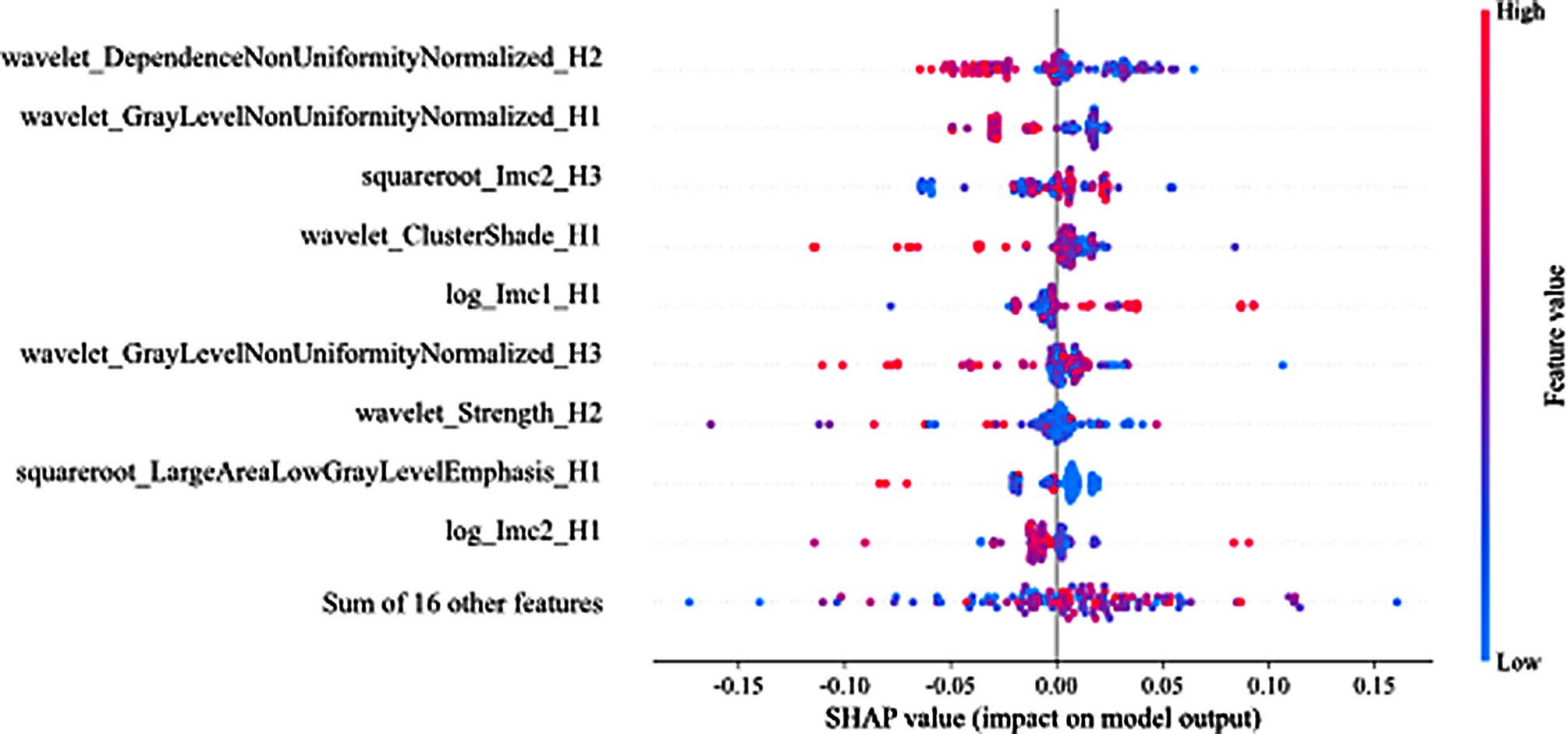
SHAP summary plot of key predictive radiomic features in the habitat-based model

SHAP (Shapley Additive exPlanations) analysis was conducted to enhance the interpretability of the ExtraTrees-based habitat model by quantifying the contribution of individual features to predictive outcomes. As illustrated in Fig. 4, SHAP values provided both local interpretability at the single-instance level and global insights into feature importance, clarifying the decision-making process of the model. Feature importance ranking revealed that descriptors derived from the H1 subregion accounted for 50% of the top 10 most influential features, while wavelet-transformed features represented approximately 50% of the entire retained feature set.

**Fig. 5:**
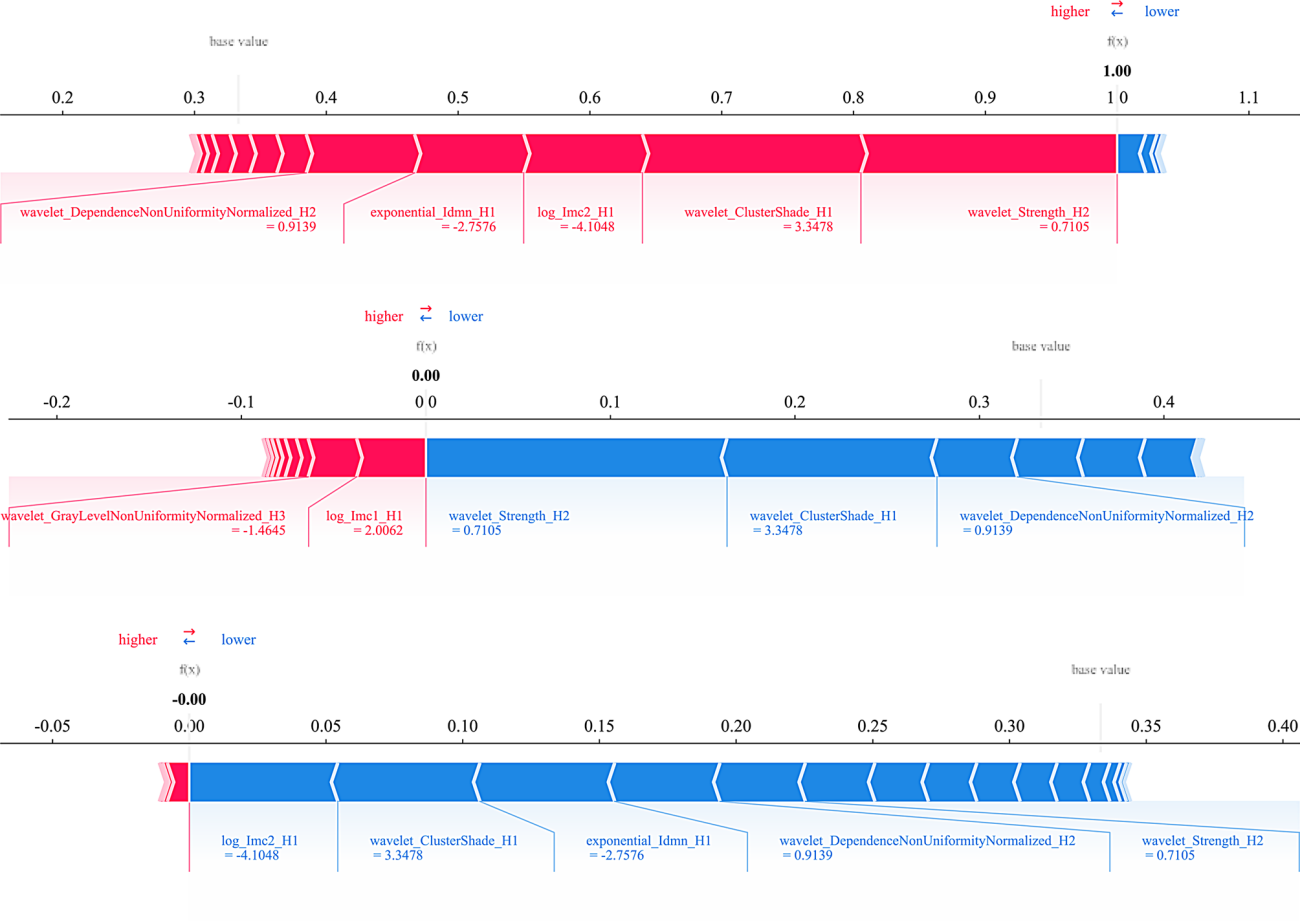
SHAP analysis of a single TRG0 sample: a force plot illustrating the SHAP values of predictive features, and a waterfall plot detailing the cumulative impact of the most significant features on the model’s final prediction

To further investigate model’s behavior, SHAP analysis was performed for individual samples with different predicted labels. Figure 5 shows the SHAP analysis results of a representative patient with TRG0 (pCR) patient in the testing cohort, including a force plot illustrating the SHAP values of individual features, and a waterfall plot detailing the cumulative contribution of the top influential features to the model’s final prediction.

### Conventional radiomics model performance

We compared the performance of SVM, RandomForest, and ExtraTrees (radiomic models) in training/testing cohorts via metrics like accuracy (Acc), AUC, sensitivity, etc.In the **training set**, ExtraTrees outperforms the others: it achieves the highest Acc (0.835), AUC (0.898), and NPV (0.916). SVM follows (Acc = 0.675, AUC = 0.784), while RandomForest has the lowest AUC (0.711).In the **testing set**, all models decline: SVM has the highest AUC (0.603), while ExtraTrees performs poorest (AUC = 0.504). Notably, ExtraTrees shows the largest drop in testing-set specificity (0.236) despite strong training-set sensitivity (0.847). Following a comprehensive evaluation of model performance, the SVM-based radiomics model was designated as the baseline model for subsequent analyses. Details of the radiomics model are summarized in Table S2.

### Clinical model performance

For the clinical signature models, ExtraTrees achieved the best overall discriminative performance, with an AUC of 0.818 (95% CI: 0.766–0.870) in the training cohort and 0.659 (95% CI: 0.551–0.768) in the test cohort, surpassing both SVM and Random Forest. Although predictive accuracy decreased slightly in the test cohort (AUC = 0.659, 95% CI: 0.551–0.768), ExtraTrees still exhibited superior stability and reliability in clinical-based risk stratification. Details of the clinical model are summarized in Table S3.

### Comparative performance evaluation of all models

In comparative analysis, the Habitat signature exhibited superior discriminative performance, with an AUC of 0.912 (95% CI: 0.875–0.949) in the training cohort and 0.817 (95% CI: 0.737–0.896) in the test cohort, surpassing both Radiomics (0.784/0.603) and Clinical (0.818/0.659). Integration of Habitat features with clinical variables further enhanced predictive accuracy, yielding an AUC of 0.916 (95% CI: 0.880–0.952) in the training cohort and 0.833 (95% CI: 0.755–0.911) in the test cohort. Table 2 summarizes the statistical data of different model performance in the training and testing sets.

The DeLong test showed that the AUC of the Habitat model was significantly higher than that of the Radiomics model in both the training and test cohorts (*P* = 0.047 and 0.024, respectively). The Combined model performed significantly better than the Radiomics model (*P* = 0.023 and 0.015, respectively) but showed no significant difference compared with the Habitat model (*P* = 0.384 and 0.453, respectively). The integration of clinical variables could optimize performance without a significant incremental benefit. Details of DeLong test are depicted in Supplementary Materials 1 (Figure S3).

**Table 2:** Performance comparison between different models in the training and testing sets

Signature	Acc	AUC	95% CI	Sensitivity	Specificity	PPV	NPV	Cohort
Clinical	0.710	0.818	0.766–0.870	0.859	0.635	0.541	0.900	Training
Clinical	0.648	0.659	0.551–0.768	0.667	0.639	0.480	0.793	Testing
Radiomics	0.675	0.784	0.728–0.840	0.871	0.576	0.507	0.899	Training
Radiomics	0.593	0.603	0.489–0.717	0.722	0.528	0.433	0.792	Testing
Habitat	0.863	0.912	0.875–0.949	0.776	0.906	0.805	0.890	Training
Habitat	0.731	0.817	0.737–0.896	0.861	0.667	0.564	0.906	Testing
Combined	0.878	0.916	0.880–0.952	0.776	0.929	0.846	0.893	Training
Combined	0.787	0.833	0.755–0.911	0.750	0.806	0.659	0.866	Testing

### TRG0 vs. others

In the TRG0 vs. TRG1–2 classification task, the Habitat model demonstrated superior discriminative capability across both cohorts. In the training set, it achieved an AUC of 0.917 (95% CI: 0.853–0.982), outperforming Clinical (0.825) and Radiomics (0.816) and approaching the Combined model (0.929). In the test cohort, the Habitat model maintained strong predictive efficacy with an AUC of 0.884 (95% CI: 0.751–1.000), exceeding both Clinical (0.848) and Radiomics (0.603), though marginally lower than the Combined model (0.929).

Collectively, these findings indicate that the Habitat model provides more robust and reliable discrimination than either Clinical or conventional radiomics models alone, underscoring its enhanced capacity to capture intratumoral heterogeneity. Moreover, integration of Habitat-derived features with clinical variables in the Combined model yielded the highest AUC values across datasets, validating the complementary value of fusing imaging-derived heterogeneity with clinical information to promote predictive performance and generalizability. Table 3 presents the classification performance of different models in TRG0 vs. TRG1–2 classification task.

**Table 3:** Performance metrics of different signatures

Signature	Accuracy	AUC	95% CI	Sensitivity	Specificity	PPV	NPV	Cohort
Clinical	0.682	0.825	0.7222 - 0.9285	0.895	0.621	0.405	0.953	training
Radiomics	0.741	0.816	0.7204 - 0.9111	0.789	0.727	0.455	0.923	training
Habitat	0.847	0.917	0.8526 - 0.9815	0.842	0.848	0.615	0.949	training
Combined	0.800	0.929	0.8680 - 0.9900	0.947	0.758	0.529	0.980	training
Clinical	0.722	0.848	0.7201 - 0.9763	1.000	0.643	0.444	1.000	test
Radiomics	0.639	0.603	0.3452 - 0.8602	0.750	0.607	0.353	0.895	test
Habitat	0.806	0.884	0.7506 - 1.0000	0.875	0.786	0.538	0.957	test
Combined	0.861	0.929	0.8463 - 1.0000	1.000	0.821	0.615	1.000	test

**Fig. 6:**
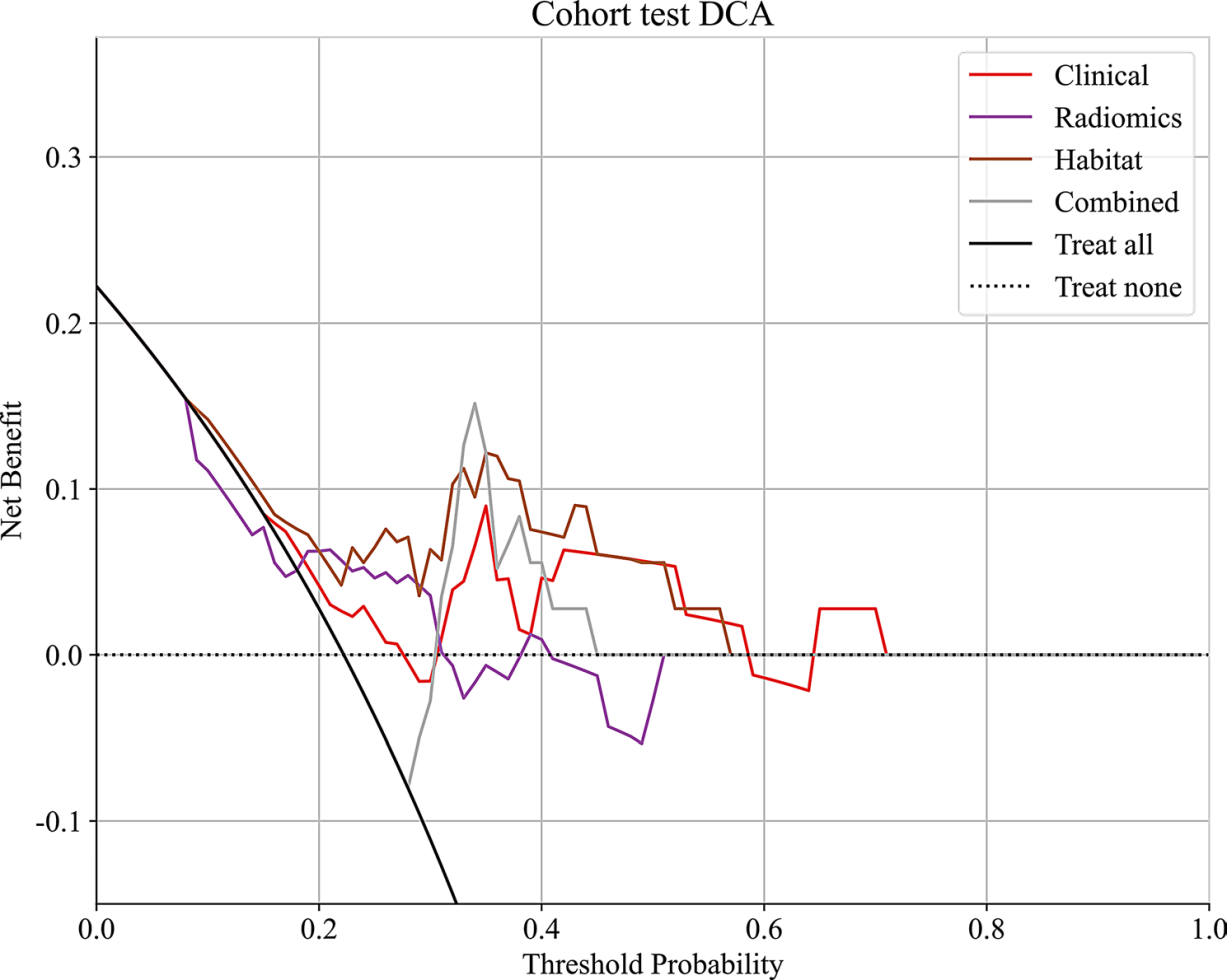
Decision curve analysis (DCA) plot of all models in the test cohort

**Fig. 7:**
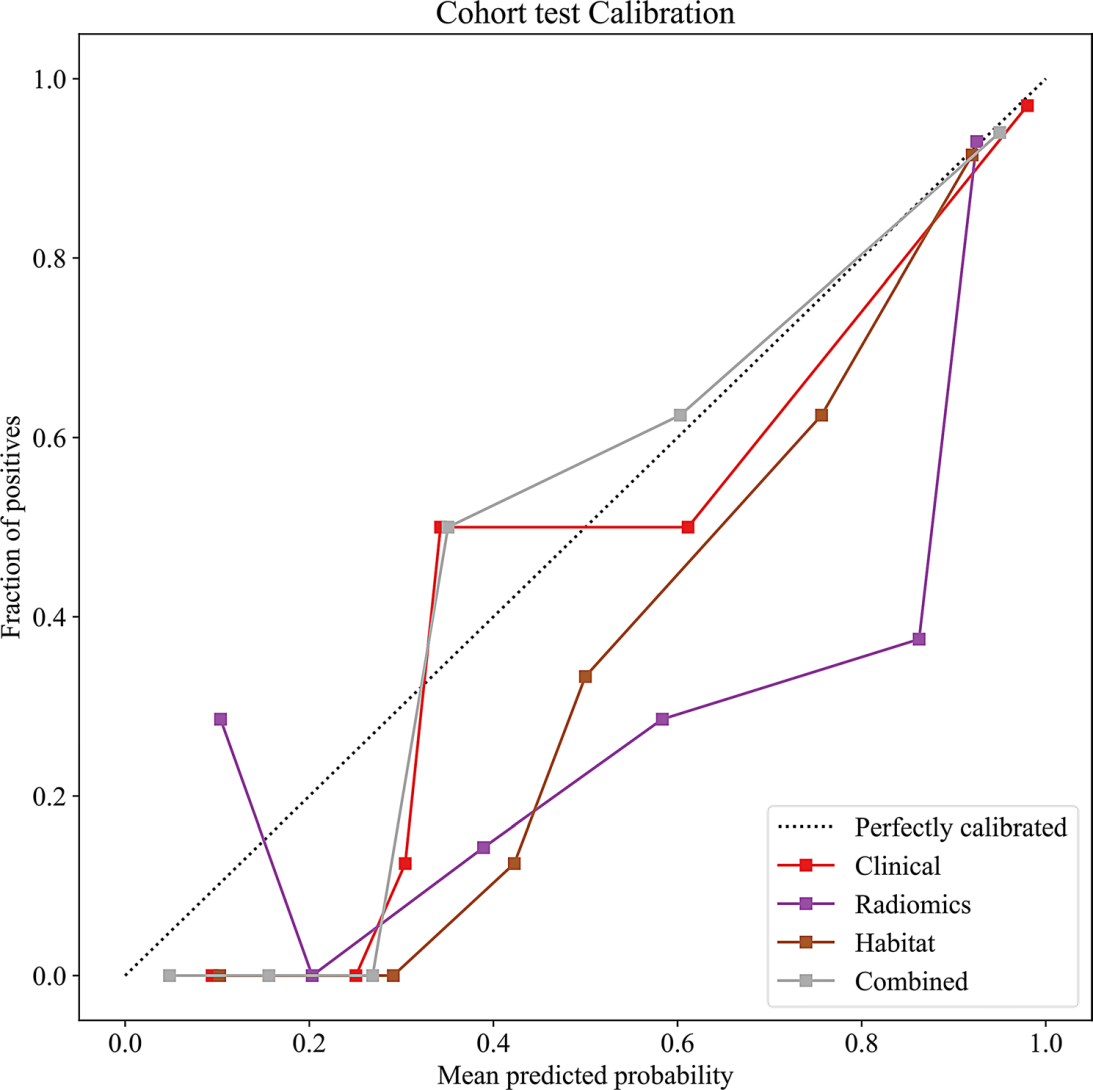
The calibration curves of all models in the test cohort

Figure 6 presents the test cohort Decision Curve Analysis (DCA) curves, with slight fluctuations due to limited sample size (*n* = 36). Within the clinically relevant TRG prediction threshold, all models showed stable calibration (HL test *P* > 0.05). DCA demonstrated that the Combined model achieved greater net clinical benefit than other models within the threshold range of 0.20–0.85, peaking at 0.45. As depicted in Fig. 7, the calibration curves demonstrated that the Radiomics model exhibited severe deviation, which not only confirms its overfitting and limited generalizability but also highlights the critical need to integrate habitat analysis into the radiomics framework. This integration is essential for improving the model’s robustness and calibration in clinical applications.

## Discussion

This study provides compelling evidence for the prognostic utility of habitat-based radiomic features in the preoperative predictive assessment of TRG in rectal cancer patients receiving nCRT. Through the extraction and quantification of 1,925 handcrafted radiomic features encompassing morphological, first-order, and texture descriptors, this investigation elucidated the capacity of quantitative imaging biomarkers to capture phenotypic heterogeneity within tumor microenvironments. Morphological features delineate geometrical and volumetric attributes of lesions, which may correlate with tumor aggressiveness, invasiveness, and therapeutic responsiveness [14, 15]. First-order intensity statistics characterize voxel-level signal distributions, reflecting fundamental biological processes [16]. Texture-based metrics further advance this framework by quantifying spatial relationships between voxels, thereby enhancing the understanding of the TME [17, 18].

The combination of mRMR and LASSO regression and 10-fold cross-validation ensures that the feature selection process is objective and operator-independent, avoiding subjective bias in manual feature selection. Missing data in clinical and imaging datasets risks losing critical biological and clinical information, reducing data mining accuracy, and increasing model overfitting. Multiple dynamic GAN approaches show great potential to address these issues by synthesizing high-quality samples and radiomic features, filling missing data with biologically plausible values, and enhancing model robustness and generalizability; we will explore integrating GAN-based data augmentation to optimize model performance in future work [19]. Despite demonstrating satisfactory discrimination in the training phase (AUC = 0.898), the conventional radiomics model exhibited a marked attenuation in generalization performance (AUC = 0.504) during testing. This decline underscores the intrinsic challenges associated with modeling multi-dimensional data structures. The integration of habitat analysis effectively delineated intratumoral heterogeneity, demonstrating superior predictive performance relative to conventional radiomics alone, thereby enhancing the robustness and reliability of TRG prediction [20–24]. Collectively, these findings underscore the potential of combining radiomic and habitat-based methodologies, supporting their continued exploration as complementary tools for refined prognostication and the development of individualized therapeutic strategies in oncologic practice.

This investigation represents a substantive advancement in the domain of medical image analysis, particularly through the comparative evaluation of predictive performance in TRG using both radiomics and habitat-based methodologies. By leveraging CT imaging data, this study juxtaposed conventional radiomic feature extraction with a habitat analysis framework to capture the intrinsic heterogeneity of tumor tissues. ROIs were meticulously segmented to enable the extraction of localized imaging features, which were subsequently subjected to voxel-level clustering to delineate distinct intratumoral subregions. This approach allowed for the quantification of spatially heterogeneous tumor characteristics—an essential determinant of tumor biology. The resultant TRG classification model integrated features derived from both radiomics and habitat analysis, underscoring the additional prognostic value conferred by habitat-based metrics relative to traditional radiomic descriptors. Notably, the combination of habitat-derived characteristics with clinical data substantially enhanced the model’s predictive accuracy, underscoring the translational potential of this integrative framework for personalized therapeutic decision-making. By elucidating the interplay between imaging-derived features and clinical outcomes, this study advances the mechanistic understanding of tumor dynamics. Such insights hold substantial implications for optimizing therapeutic strategies. SHAP analysis improves model interpretability by quantifying the contribution of each feature to TRG prediction, facilitating clinical translation [25]. The results show that wavelet texture features from the H1 proliferative zone and H3 necrotic zone are key predictors, reflecting tumor heterogeneity, cancer stem cell distribution, and necrosis extent, which are closely associated with tumor biological behavior and treatment response. Wavelet transformation better captures subtle textures and enhances sensitivity to biological changes in the tumor. Future investigations should further delineate the clinical utility of these integrative models, particularly in their capacity to predict treatment response and outcomes across heterogeneous patient populations, thereby promoting the translation of these models into precision-guided oncologic practice.

The findings of this study highlight the substantial potential of radiomics and habitat-based modeling in the predictive evaluation of TRG. The incorporation of habitat analysis into the radiomics framework demonstrably augments predictive performance. Conventional radiomic approaches, which primarily capture global tumor attributes, exhibit inherent limitations in representing the complex spatial and textural variations that underlie intratumoral heterogeneity, which are often critical for accurate TRG classification [26–29]. Conversely, the habitat model, through voxel-level clustering and localized feature extraction, effectively delineates subregional variations within tumor regions, thereby offering a more faithful depiction of tumor biology [30, 31]. Among the evaluated classifiers, the ExtraTrees model achieved superior predictive performance (AUC = 0.898 in the training cohort), outperforming both SVM and Random Forests. Nevertheless, the marked reduction in test performance observed for the radiomics model (AUC = 0.504) underscores the persistent challenges of model robustness and generalizability [32, 33]. This phenomenon suggests that the model excessively learned noise and cohort-specific patterns in the training set rather than generalizable biological features of intratumoral heterogeneity. Such overfitting may arise from the high dimensionality of global radiomic features, limited sample size, and insufficient representation of spatial heterogeneity [34]. These results highlight that conventional global radiomics is vulnerable to overfitting and lacks stability in external validation, reinforcing the importance of habitat-based local feature analysis in improving model robustness and generalization ability [35].

In addition, this study has several limitations. Although the sample size was designed in compliance with the Imaging Biomarker Standardization Initiative (IBSI) guidelines (sample-to-feature ratio ≥ 10:1) to guarantee the statistical validity of the model, this study remains a single-center retrospective investigation without external multicenter validation. Future studies will expand the multimodal sample size through multicenter collaboration and further validate the model in independent cohorts [36].Second, operator-independent deep learning (DL)-based automatic segmentation techniques (e.g., C-ENet) enable standardized, high-throughput target delineation on preoperative and follow-up tomographic images for clinical translation [37]; under clinician supervision, these methods reduce interobserver variability, optimize the radiomics workflow, and enhance model reliability and generalizability, and we plan to integrate this pipeline to build a fully automated TRG prediction system in future work. Besides, only pretreatment CT images were adopted, without longitudinal imaging data during neoadjuvant chemoradiotherapy. Delta-radiomics may help further improve the model’s predictive performance.

Accordingly, a specific and feasible plan for the clinical translation of habitat-based radiomics is provided in this study [38]. A three-stage roadmap is proposed for the clinical translation of the habitat-based radiomics model for TRG prediction: Stage 1 (0–2 years) involves large-sample multicenter validation and development of a user-friendly nomogram; Stage 2 (2–3 years) conducts a prospective trial to verify the model’s value in optimizing neoadjuvant therapy; Stage 3 (3–5 years) focuses on clinical decision support system development, protocol standardization, and health economic analysis.

## Conclusions

Habitat-based radiomics, especially when integrated with clinical variables, significantly improves the preoperative prediction of TRG in rectal cancer patients undergoing nCRT, providing a noninvasive and effective tool to advance personalized oncology for LARC. Further validation in large-scale, multicenter, independent cohorts is warranted to support the clinical translation of this approach.

## Electronic Supplementary Material

Below is the link to the electronic supplementary material.

Supplementary Material 1

Supplementary Material 2

## Data Availability

The datasets are available from the corresponding author upon reasonablerequest.

## Abbreviations

TRG: Tumor regression grading
CT: Computed tomography
ROIs: Regions of interest
SVM: Support vector machine
nCRT: Neoadjuvant chemoradiotherapy
pCR: Pathologic complete response
cCR: Clinical complete response
RC: Rectum carcinoma
ITH: Intratumor heterogeneity
VOI: Volume of interest
KNN: K-Nearest Neighbors
IBSI: Imaging biomarker standardization initiative
mRMR: Minimum redundancy maximum relevance
LASSO: Least absolute shrinkage and selection operator
SMOTE: Synthetic minority over-sampling technique
AUC: Area under the curve
SHAP: Shapley additive explanations
ANOVA: Analysis of variance
HL: Hosmer–Lemeshow
DCA: Decision Curve Analysis
DSC: Dice similarity coefficient
MSE: Mean squared error
PPV: Positive predictive value
NPV: Negative predictive value
Acc: Accuracy
