# Supplementary material for: Prediction of tumor regression grading in rectal cancer neoadjuvant chemoradiotherapy: a habitat radiomics analysis of imaging biomarker
Source: BMC Med Imaging. 2026 May 5;26:308. doi: 10.1186/s12880-026-02397-x (PMC13289560; doi:10.1186/s12880-026-02397-x)
Supplement: Supplementary file 1 — Supplementary Material 1 [file 12880_2026_2397_MOESM1_ESM.docx]

## **Supplementary**

**Appendix 1:** **habitat generation process**

Our methodology for delineating tumor habitat regions was multifaceted and involved several complex steps:

1. **Comprehensive Radiomic Feature Extraction:** This process involved extracting detailed local features from each voxel in the dataset using a $5\times5\times5$ moving window. These features encompass a variety of measurements and attributes, including intensity, texture, and other statistical properties, which are crucial for understanding the intricate details of the dataset. Such detailed insights enable more precise modeling and analysis.

In this study, 19 radiomic features were extracted from each voxel, offering a multidimensional characterization of each subregion. The 19 voxel-level features were chosen based on three principles: clinical relevance, imaging feasibility and dimensionality balance. These features included a range of shape descriptors, textural features, and first-order statistical attributes. The specific features extracted were: first-order_Entropy first-order_Mean Absolute Deviation, first-order_Median, glcm_Difference Average, glcm_Difference Entropy, glcm_Difference Variance, glcm_Imc1, glcm_Imc2, glcm_Inverse Variance, glcm_Joint Energy, glcm_Joint Entropy, glcm_SumEntropy, glrlm_Long Run Emphasis, glrlm_Run Entropy, glrlm_Run Variance, glszm_Size Zone Non Uniformity Normalized, glszm_Small Area High Gray Level Emphasis, ngtdm_Contrast, and ngtdm_Strength.

1. **In-depth Clustering Analysis:** The K-means algorithm was utilized to analyze the multidimensional feature space derived from the radiomic features. The algorithm was applied with varying numbers of cluster centers, ranging from 3 to 9, to categorize distinct habitat regions within the tumor. The performance of the clustering was evaluated using the Calinski-Harabasz score, ensuring the selection of the most statistically significant clustering arrangement.

- The K-means algorithm functions by partitioning data into K distinct clusters. It iteratively updates the centroids of these clusters to minimize the sum of squares within each cluster. The central component of the K-means algorithm is the objective function, which is optimized to achieve effective clustering.

$$J=\sum_{i=1}^{N} \sum_{k=1}^{K} w_{ik}\times\parallel x_{i}-\mu_{k}\parallel^{2}$$

- - $J$ is the objective function.
  - $N$ is the number of data points.
  - $K$ is the number of clusters.
  - $w_{ik}$ is a binary indicator (1 if data point $i$ is in cluster $k$, 0 otherwise).
  - $x_{i}$ is the ith data point.
  - $\mu_{k}$ is the centroid of cluster $k$.
  - $\parallel x_{i}-\mu_{k}\parallel^{2}$ is the squared Euclidean distance between data point $i$ and centroid $k$.

1. **Habitat Region Synthesis:** Following the clustering analysis, subregions with identical cluster IDs were amalgamated. This synthesis resulted in the formation of comprehensive habitat regions, each representing a unique microenvironmental characteristic within the tumor.


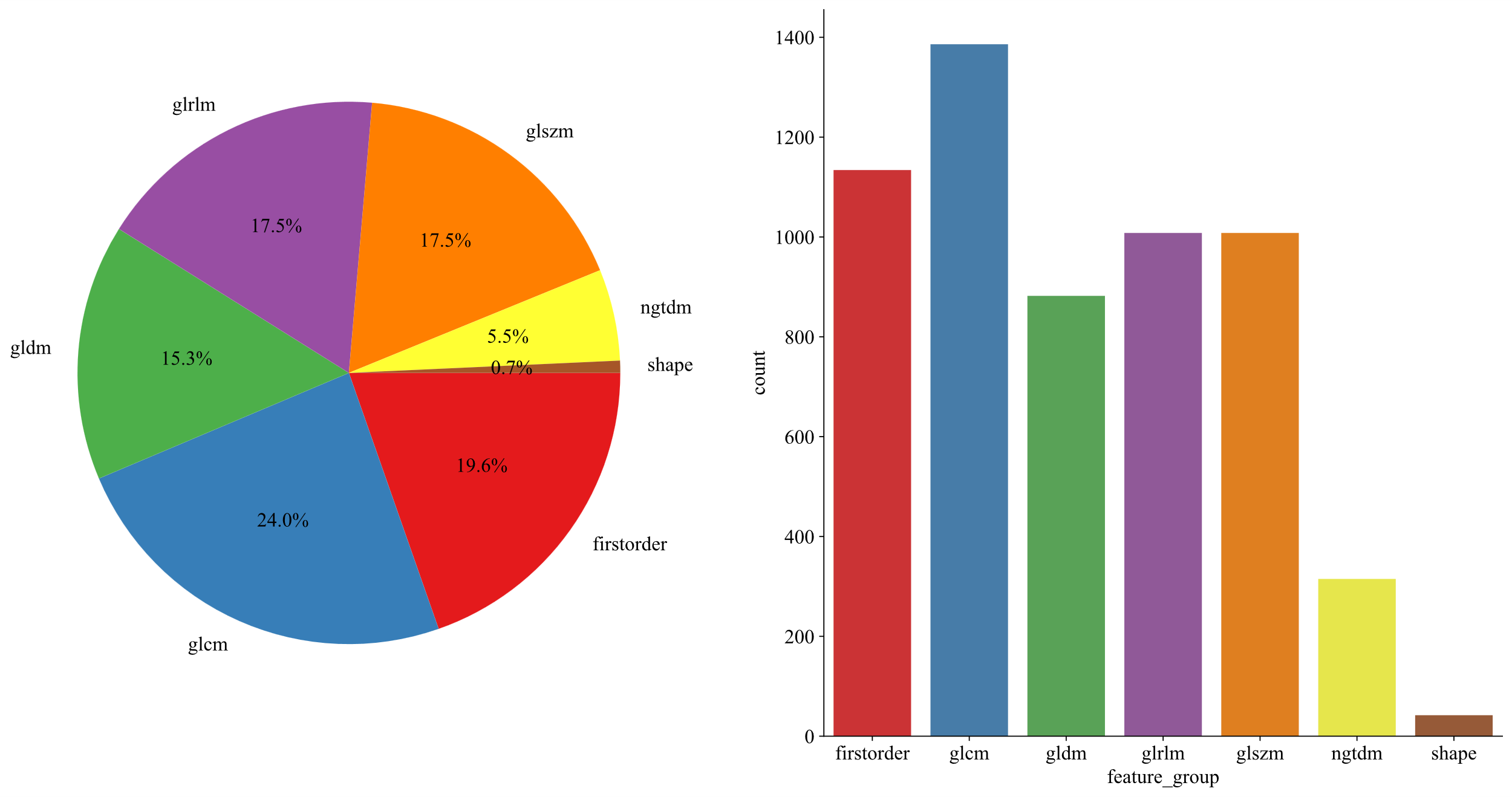


**Figure S1.** Number and ratio of handcrafted features


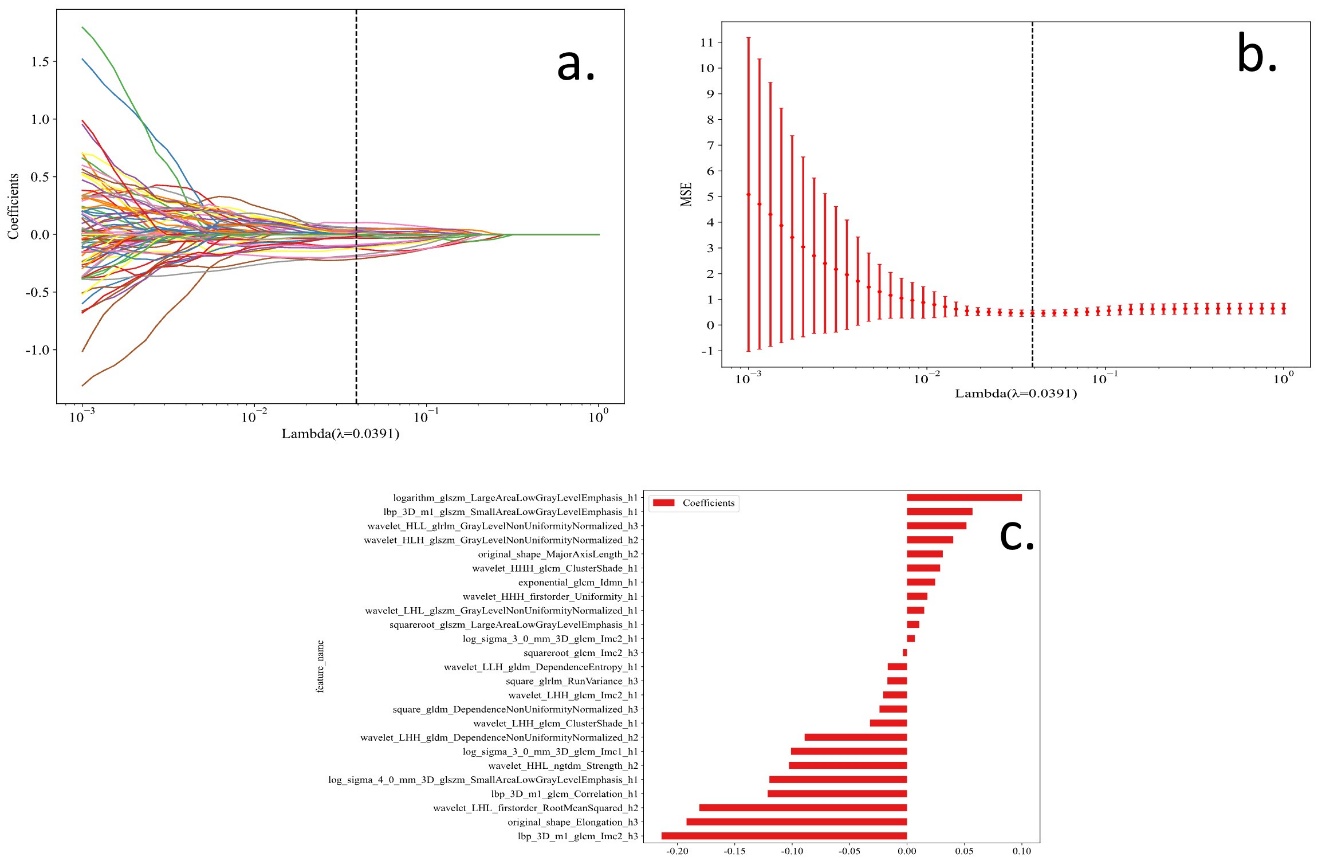


**Figure S2.** a, Coefficients of 10 fold cross validation; b, MSE of 10 fold cross validation; c,The histogram of the Rad-score based on the selected features.


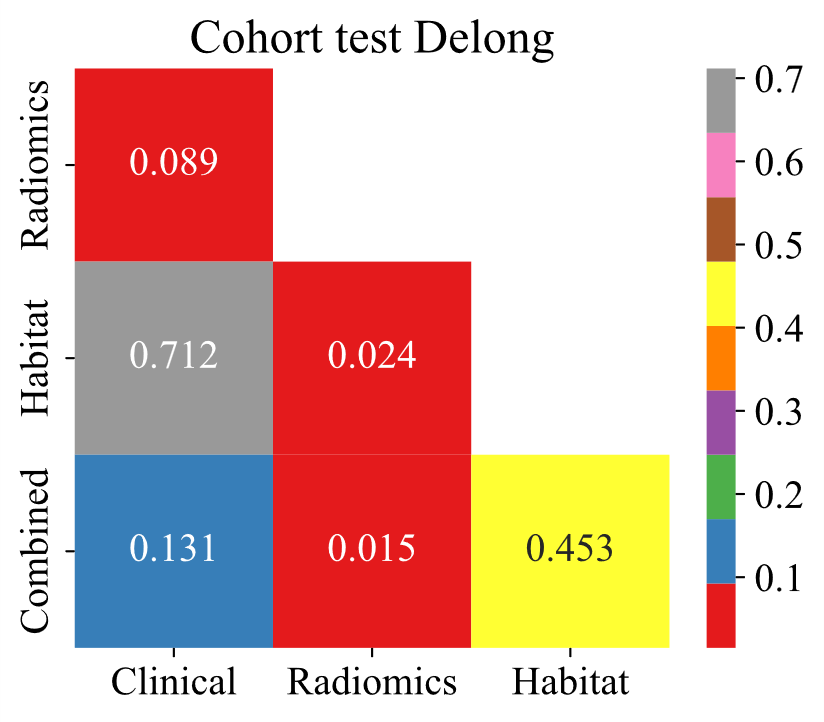


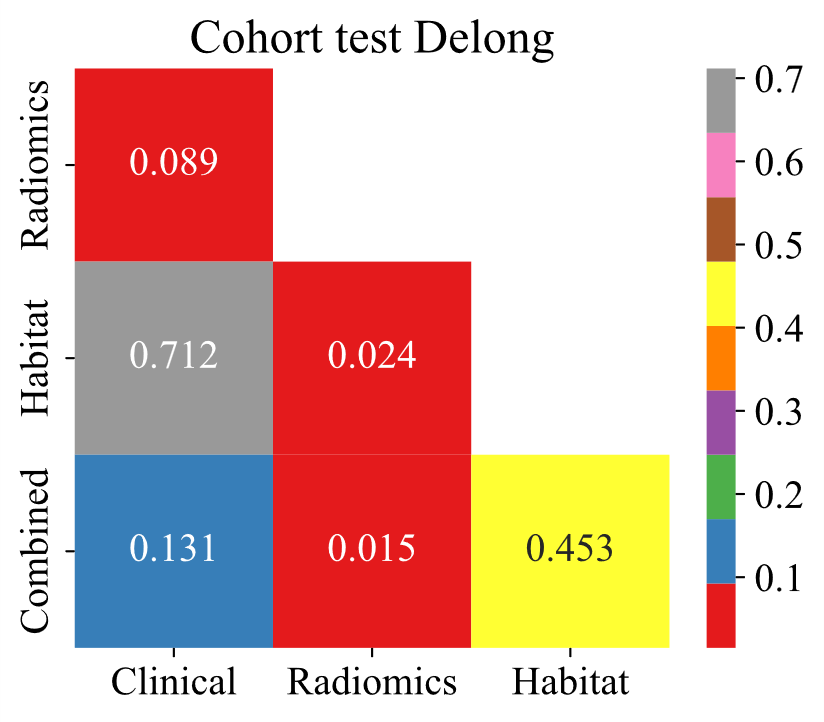


**Figure S3.** DeLong test for AUC differences between models and corresponding results

**Table S1.** Habitat Model Performance of Different Machine Learning Algorithms in the Training and Testing Sets.

| Model | Acc | AUC | 95% CI | Sensitivity | Specificity | PPV | NPV | Cohort |
| --- | --- | --- | --- | --- | --- | --- | --- | --- |
| SVM | 0.820 | 0.869 | 0.824-0.914 | 0.706 | 0.876 | 0.741 | 0.856 | Training |
| SVM | 0.611 | 0.711 | 0.610-0.813 | 0.861 | 0.486 | 0.456 | 0.875 | Test |
| RandomForest | 0.769 | 0.853 | 0.806-0.901 | 0.800 | 0.753 | 0.618 | 0.883 | Training |
| RandomForest | 0.704 | 0.582 | 0.457-0.708 | 0.444 | 0.833 | 0.571 | 0.750 | Test |
| ExtraTrees | 0.863 | 0.912 | 0.875-0.949 | 0.776 | 0.906 | 0.805 | 0.890 | Training |
| ExtraTrees | 0.731 | 0.817 | 0.737-0.896 | 0.861 | 0.667 | 0.564 | 0.906 | Test |

**Table S2.** Radiomic Model Performance of Different Machine Learning Algorithms in the Training and Testing Sets.

| Model | Acc | AUC | 95% CI | Sensitivity | Specificity | PPV | NPV | Cohort |
| --- | --- | --- | --- | --- | --- | --- | --- | --- |
| SVM | 0.675 | 0.784 | 0.728-0.840 | 0.871 | 0.576 | 0.507 | 0.899 | Training |
| SVM | 0.593 | 0.603 | 0.489-0.717 | 0.722 | 0.528 | 0.433 | 0.792 | Test |
| RandomForest | 0.733 | 0.711 | 0.643-0.780 | 0.471 | 0.865 | 0.635 | 0.766 | Training |
| RandomForest | 0.546 | 0.555 | 0.445-0.666 | 0.722 | 0.458 | 0.400 | 0.767 | Test |
| ExtraTrees | 0.835 | 0.898 | 0.857-0.938 | 0.847 | 0.829 | 0.713 | 0.916 | Training |
| ExtraTrees | 0.463 | 0.504 | 0.388-0.620 | 0.917 | 0.236 | 0.375 | 0.850 | Test |

**Table S3.** Clinical Model Performance of Different Machine Learning Algorithms in the Training and Testing Sets.

| Model | Acc | AUC | 95% CI | Sensitivity | Specificity | PPV | NPV | Cohort |
| --- | --- | --- | --- | --- | --- | --- | --- | --- |
| SVM | 0.612 | 0.648 | 0.575-0.722 | 0.682 | 0.576 | 0.446 | 0.784 | Training |
| SVM | 0.593 | 0.630 | 0.521-0.738 | 0.778 | 0.500 | 0.437 | 0.818 | Test |
| RandomForest | 0.620 | 0.682 | 0.615-0.749 | 0.718 | 0.571 | 0.455 | 0.802 | Training |
| RandomForest | 0.593 | 0.651 | 0.545-0.758 | 0.861 | 0.458 | 0.443 | 0.868 | Test |
| ExtraTrees | 0.710 | 0.818 | 0.766-0.870 | 0.859 | 0.635 | 0.541 | 0.900 | Training |
| ExtraTrees | 0.648 | 0.659 | 0.551-0.768 | 0.667 | 0.639 | 0.480 | 0.793 | Test |
